# A questionnaire survey of pharmacists regarding the clinical practice guidelines for the appropriate use of granulocyte-colony stimulating factors

**DOI:** 10.1186/s40780-018-0098-y

**Published:** 2018-01-17

**Authors:** Tetsuya Sasaki, Yasuhisa Kato, Atsushi Sato, Noriko Usui, Eishi Baba, Toshimi Takano, Nobuyuki Susumu, Kazunori Ohnishi, Hitomi Nishimoto, Katsuyuki Kiura

**Affiliations:** 1 Department of Clinical Trial Support Center, Showa University Hospital, 1-5-8 Hatanodai, Shinagawa, Tokyo, 142-8555 Japan; 2 Department of Drug Information, Showa University School of Pharmaceutical Sciences, 1-5-8 Hatanodai, Shinagawa, Tokyo, 142-8555 Japan; 3 Department of Medical Oncology, Hirosaki University Graduate School of Medicine, 5 Zaifu-cho, Hirosaki, 036-8562 Japan; 4 Department of Transfusion Medicine / Clinical Oncology and Hematology, The Jikei University Daisan Hospital, 4-11-1, Izumi-Honcho Komae-shi, Tokyo, 201-8601 Japan; 5 Department of Comprehensive Clinical Oncology, Faculty of Medical Sciences, Kyushu University, 3-1-1 Maidashi, Higashi-ku, Fukuoka, 812-8582 Japan; 6 Department of Medical Oncology, Toranomon Hospital, 2-2-2 Toranomon, Minato-ku, Tokyo, 105-8470 Japan; 7 Department of Obstetrics and Gynecology, International University of Health and Welfare, 4-3 Kozunomori, Narita-shi, Chiba, 286-8686 Japan; 8 Japanese Red Cross Aichi Blood Center, 539-3 Minami-yamaguchi, Seto, Aichi 489-8555 Japan; 9 Department of Nursing, Okayama University Hospital, 2-5-1 Shikata-cho, Okayama, 700-8558 Japan; 10 Department of Allergy and Respiratory Medicine, Okayama University Hospital, 2-5-1 Shikata-cho, Okayama, 700-8558 Japan

**Keywords:** Granulocyte Colony-stimulating factor, Febrile neutropenia, Practice guidelines, Pharmacist, Neoplasms

## Abstract

**Background:**

Clinical practice guidelines should be user-friendly and confirming their penetration rate and compliance are critical.

**Methods:**

We conducted a nationwide web-based questionnaire survey among pharmacists regarding the 2013 guidelines for the appropriate use of granulocyte-colony stimulating factors (G*-*CSFs) (version 2, published by the Japan Society of Clinical Oncology [JSCO]) between August 24 and September 6, 2015.

**Results:**

A total of 301 pharmacists responded; 96.0% belonged to hospitals and were board-certified pharmacists in oncology pharmacy (*n* = 133) and palliative pharmacy (*n* = 78). In addition, 61.5% of respondents (*n* = 185) worked for designated cancer care hospitals. The observation that 75.7% of respondents knew that the JSCO guidelines are available on the internet indicated that several pharmacists used this guideline. A high degree of usability by pharmacists was also demonstrated, as 98.0% and 51.5% of respondents, respectively, agreed with the statements “it is useful for the work of pharmacists” and “it is referred to in the actual work of pharmacists”. However, more than half of the respondents (58.4%) agreed with the phrase “there are differences from the actual work of pharmacists”.

**Conclusions:**

Their responses indicated that the respondents used the G-CSF guidelines and viewed them positively; however, the observation that about half of the respondents reported feeling that the guidelines do not match their current practice requires additional follow-up in future studies. The use of these guidelines should be routinely assessed in order to introduce novel cancer chemotherapy regimens and long-acting G-CSF in clinical practice.

## Background

Appropriate supportive care is critical for effective and safe cancer chemotherapy. In particular, febrile neutropenia (FN) is an adverse drug reaction associated with cancer chemotherapy [1] that may decrease patient quality of life (QOL) due to hospitalization or prolongation of the length of hospital stay, attenuated therapeutic efficacy due to a reduction in the relative dose intensity (RDI), impact on prognosis, and increased treatment-related mortality [2]. Thus, appropriate supportive care for FN is required. Clinical practice guidelines for granulocyte-colony stimulating factors (G*-*CSFs) are published by the American Society of Clinical Oncology (ASCO) [3, 4], the National Comprehensive Cancer Network (NCCN) (http://www.nccn.org/), and the European Organization for Research and Treatment of Cancer (EORTC) [5]. The clinical practice guidelines for the appropriate use of G-CSFs published by the Japan Society of Clinical Oncology (JSCO) in 2001 were based on the guideline requirements according to the actual situation in Japan. The guidelines were updated to version 2 in 2013 (http://jsco-cpg.jp/item/30/index.html). They recommend the use of G-CSFs based on the risk of the cancer chemotherapy regimen.

The Appraisal of Guidelines for Research and Evaluation (AGREE) (http://www.agreetrust.org/agree-ii/), which provides guidance for preparing guidelines, state that guidelines should be user-friendly, and that routine confirmation of the penetration rate and compliance are important. However, it is often unknown how guidelines are actually used in clinical practice and the accessibility, penetration rate, and compliance regarding the current JSCO guidelines have not been well assessed.

Managing FN that accompanies the use of anti-cancer drugs is important for the best practice of cancer pharmacotherapy; it is critical to assemble a team of medical care providers comprising physicians, pharmacists, and nurses. Among these team members, pharmacists play a role in providing proper cancer pharmacotherapy based on their pharmacological knowledge of general drugs, in addition to providing pharmacotherapy for cancer treatment. Therefore, we conducted a nationwide questionnaire survey among pharmacists mainly involved in cancer treatments in order to assess the penetration rate and compliance with the current guidelines in Japanese clinical practice.

## Methods

The questionnaire survey was conducted among Japanese Society of Pharmaceutical (JSPHCS)-certified oncology pharmacists (JOP), JSPHCS-certified senior oncology pharmacists (JSOP), accredited pharmacists of ambulatory cancer chemotherapy (APACC), Japanese Society of Pharmaceutical Oncology (JSPO), board-certified pharmacists in oncology pharmacy (POP), Japanese Society of Hospital Pharmacists (JSHP), board-certified pharmacists in palliative pharmacy (BCPPP), Japanese Society for Pharmaceutical Palliative Care and Sciences (JPPS), and JSCO pharmacists. We requested that they participate in this survey via e-mail. Because the members of JSHP could answer the questionnaire through a link to the members-only website, we requested that respondents who belonged to more than one society to answer through only one of them. By answering the questionnaire, the respondents were considered to have agreed to participate in this research and to agree with the publication of papers. The questionnaires were collected and counted by the JSCO administrative office and the resulting data were analyzed by researchers. This questionnaire survey-based study did not include any health care interventions. Therefore, we did not register the study protocol.

The web-based questionnaire survey was conducted for 14 days between August 24 and September 6, 2015. Text Mining Studio version 5.2 (Mathematical Systems Inc., Tokyo, Japan) was used for the analysis of the descriptions in the free description field.

## Results

### Respondent backgrounds

Answers were obtained from 301 respondents (Table 1). Among them, 96.0% (*n* = 289) were hospital pharmacists and had high-level qualifications in oncology, including board-certified POP (*n* = 133) and BCPPP (*n* = 78). Regarding the question on clinical experience, 51.8% (*n* = 156) answered “11 to 20 years”, followed by 25.9% (n = 78) who indicated “5 to 10 years”. Moreover, 61.5% (*n* = 185) of pharmacists were working at designated cancer care hospitals and 81.7% (*n* = 246) belonged to hospitals with more than 200 beds.

**Table 1:** Background of respondents

	*n* = 301
Q1. Which Medical institution do you belong to?	
Hospital	289 (96.0)^a^
Clinic	2 (0.7)
Pharmacy	6 (2.0)
Others	4 (1.3)
Q2. Do you have any certified in society (multiple choice)?	
JOP	69 (22.9)
JSOP	39 (13.0)
POP	133 (44.2)
APACC	43 (14.3)
BCPPP	78 (25.9)
None	53 (17.6)
Q3. How many duty career of clinical (year) do you have?	
≦5	13 (4.3)
5–10	78 (25.9)
11–20	156 (51.8)
21–30	45 (15.0)
≧31	9 (3.0)
Q4. Was your medical institution designated Regional Cancer Centers and Hospitals?	
Yes	185 (61.5)
No	116 (38.5)
Q5. How many hospital beds does your medical institution have?	
None	7 (2.3)
< 20	2 (0.7)
20–200	27 (9.0)
201–500	122 (40.5)
501–1000	124 (41.2)
≧1001	19 (6.3)

*JOP* JSPHCS-certified Oncology Pharmacist, *JSOP* JSPHCS-certified Senior Oncology Pharmacist, *POP* Board Certified Pharmacist in Oncology Pharmacy, *APACC* Accredited Pharmacist of Ambulatory Cancer Chemotherapy, *BCPPP* Board Certified Pharmacist in Palliative Pharmacy

^a^Number (percent)

### Use of the G-CSF guidelines in the daily work of pharmacists

Regarding the question “what do you use as a reference for the use of G-CSFs in clinical practice?”, the most frequent answer was “Clinical practice guideline” (*n* = 292) followed by “package insert of G-CSFs” (*n* = 191) (Table 2). In addition, regarding the question “how much importance do you give to the guidelines for the use of G-CSFs in clinical practice?”, the most frequent answer (62.1%, *n* = 187) was “give some important”, followed by “a high level of importance” (30.6%, *n* = 92) (Table 2). Finally, for the question “when you use the guideline for the use of G-CSF, do you use the Japanese or foreign guidelines?”, “Japanese guidelines” was the most frequent response (76.0%, *n* = 229), followed by “Japanese and foreign guidelines” (22.3%, *n* = 67) (Table 2).

**Table 2:** Use of the G-CSF guidelines in the daily work of pharmacists

	*n* = 301
Q6. What do you use as a reference for the use of G-CSFs in clinical practice?(multiple choice)	
Clinical practice guideline	292 (97.0)^a^
Guideline of ASCO	101 (33.6)
Guideline of ERTC	11 (3.7)
Guideline of NCCN	95 (31.6)
Package insert	191 (63.5)
Q7. How much importance do you give to the guideline for the use of G-CSFs in clinical practice?	
A high level of importance	92 (30.6)
Give some importance	187 (62.1)
Do not give importance much	22 (7.3)
Do not give importance at all	0 (0.0)
Q8. Do you use the Japanese or foreign guidelines?	
Japanese guidelines	229 (76.0)
Foreign guidelines	3 (1.0)
Japanese and foreign guidelines	67 (22.3)
Neither	2 (0.7)

*G-CSF* Granulocyte-Colony Stimulating Factor, *NCCN* National Comprehensive Cancer Network, *EORTC* European Organisation for Research and Treatment of Cancer, *ASCO* American Society of Clinical Oncology

^a^Number (percent)

### Use of the 2013 guidelines for the appropriate use of G-CSFs, version 2, published by the JSCO

In response to the question “do you know that the JSCO guidelines are available on the web?”, 75.7% answered “Yes”; the most frequent response to “how did you learn that the JSCO guidelines are available on a web?” was at an “academic meeting, study group meeting, workshop” (*n* = 182), followed by “website” (*n* = 124) (Table 3). Regarding the question “are the JSCO guidelines useful for the work of pharmacists?”, 55.8% (*n* = 168) answered “very useful,” and to the question “how often do you refer to the JSCO guidelines?”, the most frequent response was “sometimes” (43.5%, *n* = 131) (Table 3). In response to the question “are there any differences between the JSCO guidelines and the actual work of pharmacists?”, “somewhat different” was the most frequent response (53.8%, *n* = 162) followed by “not at all” (41.6%, *n* = 125). However, more than half of the respondents (58.4%) answered that “there are differences from the work of pharmacists” (Table 3). These differences were classified as shown in Table 3. For classification, the results of subgraph detection through co-occurrence network analysis in text mining was used as a reference (data not shown). As a result of this classification, “usage of prophylactic administration of G-CSFs” was the most frequent response (7.6%, *n* = 23), followed by “a pharmacist is not involved in the administration of G-CSFs” (6.3%, *n* = 19).

**Table 3:** Use of the 2013 guidelines for the appropriate use of G-CSFs, version 2, published by the JSCO

	*n* = 301
Q9. Do you know the JSCO guidelines are available on the web?	
Yes	228 (75.7)^a^
No	73 (24.3)
Q10. How did you learn that the JSCO guidelines are available on a web? (multiple choice)	
Academic meeting, study group meeting, workshop	182 (60.5)
Medical personnel	118 (39.2)
Academic journal, article	21 (7.0)
Website	124 (41.2)
Drug industry	44 (14.6)
Others	3 (1.0)
Q11. Are the JSCO guidelines useful for the actual work of pharmacists?	
Very useful	168 (55.8)
Useful to some extent	127 (42.2)
Not so useful	6 (2.0)
Useless	0 (0.0)
Q12. How often do you refer to the JSCO guidelines?	
Always	34 (11.3)
Usually	121 (40.2)
Sometimes	131 (43.5)
Seldom	15 (5.0)
Q13. Is there any difference between JSCO guideline and the actual work of pharmacists?	
Not at all	125 (41.6)
Somewhat different	162 (53.8)
Considerably difference	13 (4.3)
Completely different	1 (0.3)
Q14. To the Q13. which chose “it is totally considerably slightly different with the difference with the difference” by a question. What kind of difference was it?	
Usage of the prophylaxic administration of G-CSFs	23
A pharmacist is not involved in the administration of G-CSFs	19
It is difficult to fit a real patient	15
Usage of no exothermicity neutropenia	11
There is not consensus in the nosocomial G-CSFs usage	9
The update of guidelines is late	5
It is different from the usage of the package insert	5
About the risk classification of the cancer chemotherapy regimen	4
Usage of the remedial dosage	4
Others	11

*JSCO* Japan Society of Clinical Oncology, *G-CSF* Granulocyte-Colony Stimulating Factor

^a^Number (percent)

### Suggestion for the use of G-CSFs in individual cases

For cancer chemotherapy regimens with a combination therapy of docetaxel and cyclophosphamide (TC therapy) for the treatment of breast cancer, with an FN incidence > 20%, the respondents were likely to suggest the primary prophylactic administration of G-CSFs (“I recommend of primary prophylactic administration of G-CSFs”: 29.7%) (Table 4). However, when a combination therapy of cisplatin and tegafur/gimeracil/oteracil (S-1) was used for the treatment of advanced and recurrent gastric cancer and a combination therapy of irinotecan, oxaliplatin, and 5-fluorouracil (FOLFOXIRI) was used for the treatment of advanced and recurrent colorectal cancer, with an FN incidence of below 20%, the respondents were unlikely to suggest the primary prophylactic administration of G-CSFs (“I recommend of primary prophylactic administration of G-CSFs”: 0.5% and 12.6%, respectively) (Table 4). Regarding the suggestion for the secondary prophylactic administration of G-CSFs for squamous cell lung cancer patients treated with docetaxel alone, 43.4% and 39.3% of the respondents indicated “I use” and “I do not use”, respectively (Table 5).

**Table 4:** Suggestion for the use of G-CSFs in individual cases. Primary prevention dosage of G-CSFs

	Q16. TC for breast cancer^a^	Q17. CDDP + S-1 for gastric cancer^b^	Q18. FOLFOXIRI for colorectal cancer^c^
	*n* = 145	*n* = 187	*n* = 183
I recommend of primary prophylactic administration of G-CSFs	43 (29.7)^d^	1 (0.5)	23 (12.6)
After Grade 4 neutropenia developed, I recommend G-CSFs	48 (33.1)	63 (33.7)	70 (38.2)
After FN developed, I recommend G-CSFs	54 (37.2)	66 (35.3)	56 (30.6)
I do not recommend G-CSFs	0 (0.0)	57 (30.5)	34 (18.6)

*TC* Docetaxel and Cyclophosphamide, *CDDP* Cisplatin, *S-1* Tegafur, gimeracil, and oteracil, *FOLFOXIRI* Irinotecan, oxaliplatin, and 5-fluorouracil, G-*CSF* Granulocyte-Colony Stimulating Factor, *FN* Febrile Neutropenia

^a^A 67-year-old woman. You plan to treat with TC therapy (docetaxel 75 mg / m^2^ and cyclophosphamide 600 mg / m^2^, administered every 3 weeks) as postoperative chemotherapy for breast cancer. What kind of suggestion do you do as a pharmacist?

^b^A 70-year-old man. As first-line chemotherapy for unresectable gastric cancer, you plan to treat with S-1 and cisplatin combination therapy. What kind of suggestion do you do as a pharmacist?

^c^A 70-year-old woman. You plan to treat with irinotecan, oxaliplatin and 5-fluorouracil (FOLFOXIRI) therapy as first-line chemotherapy for unresectable colon cancer. What kind of suggestion do you do as a pharmacist?

^d^Number (percent)

**Table 5:** Suggestion for the use of G-CSFs in individual cases. Secondary prevention dosage of G-CSFs

	Q15. DTX for squamous cell lung cancer^a^
	*n* = 168
I recommend of secondary prophylactic administration of G-CSFs	73 (43.4)^b^
After Grade 4 neutropenia developed, I recommend G-CSFs	23 (13.7)
After FN developed, I recommend G-CSFs	6 (3.6)
I do not recommend G-CSFs	66 (39.3)

*DTX* Docetaxel, *G-CSF* Granulocyte-Colony Stimulating Factor, *FN* Febrile Neutropenia

^a^A 78-year-old man. You diagnosis of lung squamous cell carcinoma and started docetaxel alone therapy, but complication of febrile neutropenia was appeared. You confirmed a good tumor reduction effect, so you plan to treat the second course. What kind of suggestion do you do as a pharmacist?

^b^Number (percent)

Regarding the usage of G-CSF during concurrent chemoradiotherapy (cisplatin 40 mg/m^2^ on days 1, 8, 15, 22, 29, and 36) in patients with stage IIB cervical cancer, “use of G-CSFs is suggested only for high-risk patients” was the most frequent response (30.7%, *n* = 35) (Table 6).

**Table 6:** Suggestion for the use of G-CSFs in individual cases. Usage of the GCSFs preparation for cervical cancer

	Q19. CCRT for cervical cancer^a^
	*n* = 114
I recommend G-CSFs on the following day of chemotherapy	6 (5.2)^b^
After Grade 4 neutropenia developed, I recommend G-CSFs	23 (20.2)
After FN developed, I recommend G-CSFs	26 (22.8)
I recommend G-CSFs to only high risk patients (Less than neutrophilic 100/mm^3^continues more than 10 days and/or fungal infectious disease)	35 (30.7)
I do not recommend G-CSFs	24 (21.1)

*CCRT* Concurrent Chemoradiotherapy, *G-CSF* Granulocyte-Colony Stimulating Factor, *FN* Febrile Neutropenia

^a^A 30-year-old woman. We plan to concurrent chemoradiotherapy (cisplatin 40 mg/m^2^ on days 1, 8, 15, 22, 29, and 36) in patients with stage IIB cervical cancer (squamous cell carcinoma)

^b^Number (percent)

### Prophylactic administration and adverse drug reactions to G-CSFs

Regarding the use of pegfilgrastim during 2-week interval chemotherapy, “I suggest that pegfilgrastim be used on the following day of chemotherapy and on the following day of chemotherapy in the next cycle” was the most frequent response (56.7%, *n* = 93) (Table 7). In response to the question “is G-CSF used as primary prophylaxis at your institute?”, 55.3% (*n* = 162) indicated that “G-CSF is used as primary prophylaxis only during chemotherapy aimed at achieving a cure or prolonging survival time” (Table 7).

**Table 7:** Prophylactic administration and adverse drug reactions to G-CSFs

Q20. Regarding the use of pegfilgrastim during 2-week interval chemotherapy, what kind of suggestion do you do? (*N* = 164)	
Use pegfilgrastim on the day of chemotherapy	2 (1.2)
Use pegfilgrastim on the following day of chemotherapy	93 (56.7)
Use pegfilgrastim on the following day of chemotherapy. I administer chemotherapy one day later of the next cycle, and use pegfilgrastim on the following day of chemotherapy	8 (4.9)
Do not use pegfilgrastim	61 (37.2)
Q21.What explanation or suggestion do you provide for bone pain, an adverse drug reaction, in the use of G-CSFs? (*N* = 187)	
Do not explain about adverse events of G-CSFs to a patient	34 (18.2)
Have a medical examination in the occurrence of bone pain	67 (35.8)
Prescribe oral NSAIDs in advance of the occurrence of bone pain	85 (45.5)
Prescribe oral antihistaminic drugs in advance in the occurrence of bone pain	1 (0.5)
Q22. Are G-CSFs used as primary prophylaxis at your institute? (*N* = 293)	
We used G-CSFs only during chemotherapy aimed at the symptom palliation	39 (13.3)
We used G-CSFs only during chemotherapy aimed at achieving a cure or prolonging survival time	162 (55.3)
We do not use G-CSFs as primary prophylaxis	92 (31.4)

*G-CSF* Granulocyte-Colony Stimulating Factor, *NSAIDs* Non-Steroidal Anti-Inflammatory Drugs

When asked “what explanation or suggestion do you provide for bone pain, an adverse drug reaction, in the use of G-CSFs?”, the most common response (45.5%, *n* = 85) was “I will suggest prescribing oral NSAIDs in advance of the occurrence of bone pain” (Table 7).

## Discussion

In this questionnaire survey study of pharmacists engaged in cancer treatment nationwide, answers were obtained from 301 respondents. To our knowledge, the present study is the first and largest questionnaire regarding G-CSFs guidelines in Japan. Of all respondents, 82.4% were certified, accredited, or senior pharmacists in the field of cancer and palliative care; thus, responses were obtained from pharmacists who are mostly involved in cancer treatment across Japan, suggesting that the findings reflect the current use of G-CSFs at cancer treatment specialty hospitals throughout Japan.

User penetration rate and compliance are both important items for the evaluation of clinical practice guidelines. The penetration rate of the guideline was evaluated by the responses to question number 9. In this survey, 75.7% of participants recognized the availability of the JSCO guidelines on the web, thus indicating that the guidelines are widely accepted. In a prospective observational study conducted in Iran, 63.7% of prescriptions are in compliance with ASCO guidelines [6]. A typical German sample survey reported that 85.1% of physicians adhere to G-CSF EORTC guidelines [7]. Our current results support these previous findings. The high usability of the guidelines was demonstrated by the observation that 98.0% of the respondents answered that the guidelines are “useful for the work of pharmacists” and 51.5% replied that the guidelines are “referred in the work of pharmacists” (Table 3). The usefulness of these guidelines as work guidelines for pharmacists was indicated by the observations that 98.0% reported the guidelines to be "beneficial to the work of pharmacists" and that 51.5% indicated that the guidelines are "being referred to in the pharmacist's work" (Table 3). However, many respondents reported that "there are differences from the pharmacist's work" (58.4%), citing “prophylactic administration” as the reason for these differences. This is because the experience of PEGylated long-acting G-CSFs since their introduction in Japan in 2014 is inadequate and there is a large gap between clinical practice and the current guidelines. The second most frequent answer was that "Pharmacists are not involved in the administration of G-CSFs". In general, patient characteristics (age, complications, and general condition) are also important considerations in cancer chemotherapy regimens and help to determine whether G-CSFs are used. Therefore, in many cases, the clinical judgment of the physicians may be given priority (Table 3). However, aggressive drug therapy should be under the guidance of a pharmacist. Pharmacists have knowledge regarding pharmaceutical science not only for anticancer drugs but also for general medicine. Therefore, it is important for cancer board-certified pharmacists to be included in discussions with doctors and medical staff regarding hospitalized and outpatient cancer patients. In addition, it is useful to periodically survey the actual clinical situation and to provide feedback to doctors on the proper use of G-CSFs.

Both national and international guidelines recommend the use of primary prophylaxis for cancer chemotherapy regimens with an FN incidence rate > 20% [3–6]. When the cancer chemotherapy regimen (TC therapy for breast cancer) had an FN incidence rate > 20%, it was highly likely that the pharmacist would propose the use of primary prophylactic G-CSFs (29.7%) (Table 4). In contrast, the use of primary prophylactic G-CSFs was low (0.5% and 12.6%, respectively) in cancer chemotherapy regimens including cisplatin and S-1 therapy for gastric cancer and FOLFOXIRI therapy for colorectal cancer with FN incidence rates below 20% (Table 4). As mentioned above, more pharmacists were likely to suggest the primary prophylactic administration of G-CSFs according to the treatment strategy recommended by the JSCO guidelines. In contrast, the response differed with respect to the secondary prophylactic administration of G-CSFs (Table 5). This difference is likely because the JSCO guidelines’ recommended grade for the secondary prophylactic administration of G-CSFs is "C2 (the evidence is not known clearly)". As indicated above, the use of G-CSFs as a secondary prophylactic should be decided in individual patients.

The collection rate of questionnaires is one of the factors used to evaluate the reliability of the results. However, we failed to determine the collection rate in this survey. This is because we requested participation from five academic societies whose expert pharmacists engaged mainly in cancer treatment; among them, four societies (JSPHCS, JSPO, JPPS, and JSCO) directly asked their members to participate via e-mail. However, the questionnaire was linked to a members-only website of the JSHP because e-mail distribution was difficult for this society. Therefore, the collection rate was not calculated because the total number of responses requested was uncertain.

## Conclusions

The results of this questionnaire survey indicate that the guidelines on the proper use of G-CSFs issued by the JSCO have been widely accepted among pharmacists and are very useful. However, some negative opinions were observed. While clinical practice guidelines are generally revised every 3 years [8], it may be necessary to periodically revise these guidelines owing to the introduction of new anticancer drug therapy and long-acting G-CSFs.

## Abbreviations

AGREE: Appraisal of Guidelines for Research and Evaluation
APACC: Accredited pharmacists of ambulatory cancer chemotherapy
ASCO: American Society of Clinical Oncology
BCPPP: Board certified pharmacists in palliative pharmacy
EORTC: European Organization for Research and Treatment of Cancer
FN: Febrile neutropenia
G*-*CSF: Granulocyte-colony stimulating factor
JOP: JSPHCS-certified oncology pharmacists
JPPS: Japanese Society for Pharmaceutical Palliative Care and Sciences
JSCO: Japan Society of Clinical Oncology
JSCO: Japan Society of Clinical Oncology
JSHP: Japanese Society of Hospital Pharmacists
JSOP: JSPHCS-certified senior oncology pharmacists
JSPHCS: Japanese Society of Pharmaceutical
JSPO: Japanese Society of Pharmaceutical Oncology
NCCN: National Comprehensive Cancer Network
POP: Pharmacists in oncology pharmacy
QOL: Quality of life
RDI: Relative dose intensity
